# Papilloedema is the point: shared downstream biological mechanisms do not establish a migraine–idiopathic intracranial hypertension disease continuum

**DOI:** 10.1093/braincomms/fcag415

**Published:** 2026-10-30

**Authors:** Andreas Yiangou, Alexandra J Sinclair

**Affiliations:** Metabolism and Systems Science, School of Medical Sciences, College of Medicine and Health, University of Birmingham, Birmingham B15 2TT, UK; Department of Neurology, University Hospitals Birmingham NHS Foundation Trust, Birmingham B15 2GW, UK; Metabolism and Systems Science, School of Medical Sciences, College of Medicine and Health, University of Birmingham, Birmingham B15 2TT, UK; Department of Neurology, University Hospitals Birmingham NHS Foundation Trust, Birmingham B15 2GW, UK

## Abstract

This scientific commentary refers to ‘Papilledema is not the point: intracranial pressure dysregulation as a common pathway in migraine and idiopathic intracranial hypertension’, by Braca *et al*. (https://doi.org/10.1093/braincomms/fcag259).

## Introduction

In their recent article in *Brain Communications,* Braca *et al*.^1^ report a retrospective MRI/MRV study comparing 125 patients with migraine, 36 with idiopathic intracranial hypertension (IIH) and 37 healthy controls, suggesting progressively greater venous sinus narrowing across these groups. Using an extended combined conduit score (ECCS) to quantify venous sinus narrowing, they found higher scores in migraine than controls and substantially higher scores in IIH. They also assessed other radiological features associated with raised intracranial pressure (ICP), with empty sella more frequent in migraine than controls and these features overall most prominent in IIH. From these imaging associations, they propose that ICP regulation may represent a shared pathogenetic substrate linking migraine and IIH, with papilloedema reflecting disease severity rather than defining a distinct disease process. This is a notable and provocative hypothesis, particularly given the substantial phenotypic overlap between headache attributed to IIH and migraine.

There is compelling evidence that migraine and IIH converge upon shared headache biology. Experimental calcitonin gene-related peptide (CGRP) administration provokes migraine-like headache in IIH with associated neuroimmune activation, supporting trigeminovascular involvement.^2,3^ However, shared downstream mechanisms do not establish shared upstream disease pathogenesis and radiological features associated with raised ICP are not equivalent to demonstration of raised ICP. The critical question is whether the imaging observations of Braca *et al*. demonstrate ICP dysregulation upstream of migraine and thereby support a single disease continuum. We propose an alternative framework in which distinct upstream disease processes converge upon shared downstream trigeminovascular and neuroimmune mechanisms, with sustained raised ICP providing one route to migraine-like headache biology in IIH (Fig. 1).

**Figure 1:**
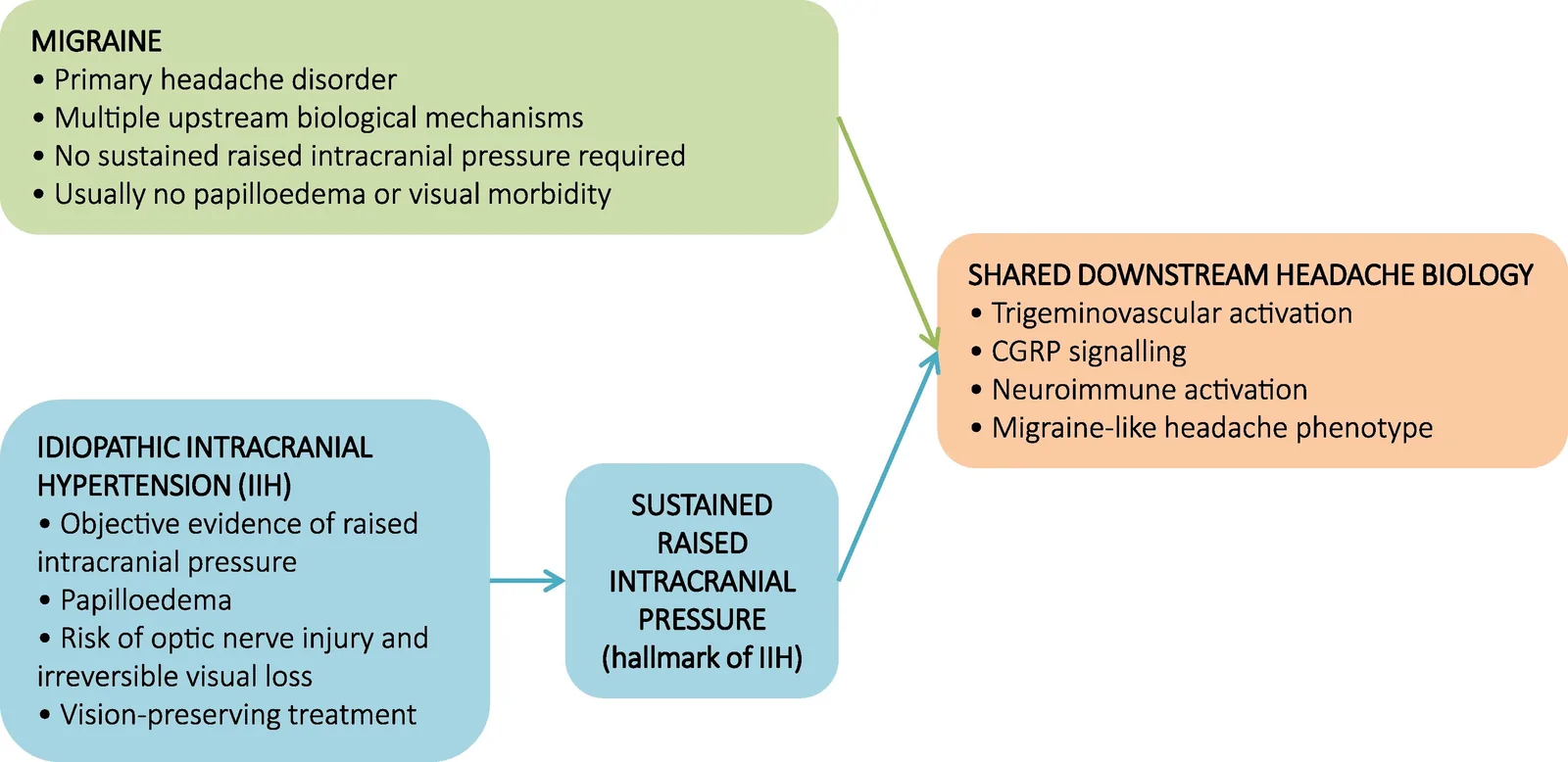
**Proposed conceptual framework.** The figure distinguishes shared downstream biological mechanisms from distinct upstream disease mechanisms in migraine and idiopathic intracranial hypertension.

## Shared biology, imaging and intracranial pressure: association is not mechanism

Braca *et al*. cite therapeutic overlap between migraine and IIH, including responses to topiramate and lumbar puncture (LP), as supporting a shared pressure-related pathophysiology. However, therapeutic overlap is difficult to interpret mechanistically: topiramate has antimigraine actions beyond carbonic anhydrase inhibition, acetazolamide is not established for IIH headache alone and improvement following LP is neither universal nor sustained.^4,5^ Therapeutic overlap therefore does not establish pressure-dependent migraine biology. The authors also invoke elevated dural sinus pressure as a ‘universal mechanism’ of raised ICP. This concept derives principally from the cited Karahalios study of ten patients nearly three decades ago, whereas contemporary understanding of IIH encompasses interacting metabolic, hormonal, CSF-secretory and venous mechanisms.^6,7^

The central methodological issue is the extrapolation from an imaging phenotype to presumed ICP dysregulation. Although the imaging scores (ECCS) differed between groups, distributions substantially overlapped between controls and migraine (median 2 (1–3), 3 (2–4) and 3 (3–4) in controls, migraine without aura and migraine with aura, respectively), whereas IIH showed considerably greater separation (6 (5–6)). The ECCS ≥3 threshold used to dichotomize venous stenosis should remain explicitly exploratory. It was derived by receiver operating characteristic (ROC) analysis with internal split-sample validation, but without external validation or sensitivity testing of alternative thresholds; the authors themselves acknowledge this limitation. Dichotomization may obscure the underlying distributions, while neither analysis establishes altered ICP or a migraine–IIH continuum.

More fundamentally, ICP was not measured in the migraine cohorts: the study demonstrates an imaging phenotype, not intracranial hypertension in migraine.^8-10^ Importantly, migraine-like headache can occur independently of changes in mean ICP: in a randomized double-blind placebo-controlled experimental study, CGRP provoked attacks in 71% of participants with IIH versus 18% after placebo, despite no change in mean ICP.^3^ Potential confounding also limits interpretation of the between-group imaging differences. BMI was available only for the IIH cohort, preventing adjustment across groups, while controls were predominantly male despite the marked female predominance of migraine and IIH. The authors appropriately acknowledge both limitations.

The study found no association between imaging abnormalities and headache frequency and the authors interpret this as suggesting that impaired ICP regulation represents a background predisposition to migraine, with individual susceptibility determining whether and how frequently headache develops. However, a negative cross-sectional association cannot establish that these imaging abnormalities precede migraine, confer susceptibility or reflect altered ICP. To support this interpretation, the authors draw an analogy with CGRP and pituitary adenylate cyclase-activating polypeptide provocation studies, in which these peptides trigger attacks preferentially in people with migraine, suggesting that an underlying susceptibility is required for headache expression. The analogy does not, however, establish ICP dysregulation as that susceptibility: in provocation studies the biological perturbation is directly administered and its temporal relationship with headache observed, whereas here altered ICP is inferred from imaging rather than demonstrated.^2,3^

On this basis, Braca *et al*. propose that impaired intracranial volume and pressure regulation is a ‘necessary, though not sufficient’ condition for migraine development, with an additional individual predisposition determining headache expression. This is a substantially stronger claim than the data support. Demonstrating necessity would require evidence that migraine does not develop without ICP dysregulation and that this abnormality is consistently present when migraine occurs, alongside direct evidence that the imaging phenotype corresponds to altered ICP. The distinction between observation and interpretation is summarized in Table 1.

**Table 1:** Distinguishing observation from interpretation

Observation	Proposed interpretation	Alternative interpretation
Higher ECCS in migraine than controls	Migraine–IIH continuum	Shared imaging features do not establish shared disease
Novel ECCS ≥3 threshold	Pathological venous abnormality	Data-derived threshold requiring independent validation
MRI findings more frequent in migraine	Occult ICP dysregulation	MRI findings are supportive in IIH, not diagnostic and require clinical context
Migraine-like headache in IIH	Shared disease spectrum	Shared trigeminovascular biology with distinct underlying diseases
Similar outcomes across BMI groups in IIH	Obesity acts through altered fluid dynamics	Prognostic studies do not establish disease mechanisms
Papilloedema absent in some patients	Papilloedema is not central	Papilloedema remains central to diagnosis, visual risk and management in IIH

ECCS: Extended Combined Conduit Score, IIH: Idiopathic intracranial hypertension, ICP: Intracranial pressure

## Papilloedema remains central in IIH

Braca *et al*. interpret the greater severity and bilaterality of venous sinus narrowing in IIH as suggesting that papilloedema marks the severe end of an ICP-dysregulation spectrum rather than distinguishing a separate disease process. However, papilloedema is optic disc swelling secondary to raised ICP and the principal clinical marker through which IIH threatens vision.^4,11^ Although susceptibility, magnitude and duration of ICP elevation may modify its development and severity, this does not make papilloedema independent of ICP. Without demonstrating raised ICP in the migraine cohorts, the present data cannot establish migraine without papilloedema as a milder manifestation of the same disease process. Current diagnostic frameworks recognize IIH without papilloedema (IIHWOP) but deliberately require objective evidence of raised ICP together with stringent additional criteria when papilloedema is absent.^4,11^ This distinction is important because headache phenotype and individual MRI findings alone lack sufficient specificity for IIH.^4,8-11^

Neuroimaging should therefore remain supportive rather than a substitute for clinical and physiological diagnosis. MRI features associated with raised ICP including empty sella, optic nerve sheath distension, posterior globe flattening and transverse sinus stenosis can frequently occur outside IIH, including in healthy subjects.^8-10^ Incidental MRI signs of intracranial hypertension are considerably more frequent than papilloedema, while IIHWOP and normative imaging studies emphasize the importance of combinations of signs and clinical context.^8-10^ Extending an ICP-based disease model to patients with migraine on the basis of supportive or incidental MRI/MRV findings risks diagnostic overreach, particularly when papilloedema is absent.^4,11^ This distinction is clinically consequential: IIH management aims to preserve vision, modify disease and treat headache morbidity.^4^ Papilloedema is therefore ‘the point’ because it identifies the vision-threatening manifestation that fundamentally changes clinical management.

## Clinical implications: association should not drive intervention

Lumbar puncture should not become a means of validating non-specific imaging abnormalities in otherwise typical migraine. Therapeutic LP provides neither reliable nor sustained headache benefit in IIH and response is unrelated to opening pressure whilst post-LP headache exacerbation is common.^4,5^ Similarly, in the IIH Treatment Trial, acetazolamide reduced ICP and papilloedema without significantly improving headache disability versus placebo.^12^ Thus, ICP reduction does not necessarily translate into improvement of migraine-like headache.

Conversely, in patients with IIH whose headaches responded to erenumab, papilloedema recurred without headache recurrence.^13^ This dissociation supports treating migraine-like headache according to phenotype while independently monitoring and treating vision-threatening IIH. Pressure-lowering intervention remains essential when required to protect vision, whereas established pharmacological approaches, including CGRP-pathway therapies, provide a more targeted and less invasive approach when headache itself is the therapeutic target. The proposal that impaired venous outflow and ICP regulation represents a novel therapeutic target for migraine is therefore very premature. Translation would require prospective evidence linking the imaging phenotype to directly measured ICP and, ultimately, randomized trial evidence that modifying the proposed mechanism improves clinically meaningful migraine outcomes.

The discussion of obesity illustrates a similar transition from association to causality. Braca *et al*. propose obesity principally as a fluid-dynamic risk factor for papilloedema rather than a dysmetabolic contributor to IIH, although BMI was unavailable outside the IIH cohort and neither central venous pressure nor ICP was measured. Contemporary evidence supports a multifactorial metabolic, hormonal, CSF and venous model.^6^ The cited Thaller *et al*. study of visual outcomes across BMI groups is prognostic, not mechanistic, and cannot establish how obesity contributes to disease initiation.^14^ Collectively, these observations suggest that ICP may activate or modulate migraine biology in IIH, but headache expression is not simply coupled to contemporaneous ICP, favouring biological convergence rather than disease continuity (Fig. 1).

## An important hypothesis requiring the right experiment

Braca *et al*.’s imaging findings establish an association but not altered ICP, causality or a migraine–IIH disease continuum. Testing this hypothesis requires prospective, appropriately sex- and BMI-matched cohorts, prespecified and independently validated imaging measures and direct ICP assessment where clinically and ethically appropriate. Longitudinal studies should establish whether venous abnormalities precede migraine and correlate with objectively demonstrated ICP dysregulation. Ultimately, causality requires perturbation: modifying the proposed mechanism should alter clinically meaningful migraine outcomes.

Shared downstream trigeminovascular and neuroimmune mechanisms provide a plausible explanation for overlapping headache phenotypes without requiring migraine and IIH to represent different severities of the same disease. Papilloedema remains the point because it identifies the clinically consequential intersection between sustained intracranial hypertension, optic nerve injury and preventable visual loss.

## Data Availability

Data sharing is not applicable to this article as no new data were created or analysed.
